# The actual and ideal indoor soundscape for work, relaxation, physical and sexual activity at home: A case study during the COVID-19 lockdown in London

**DOI:** 10.3389/fpsyg.2022.1038303

**Published:** 2022-12-29

**Authors:** Simone Torresin, Eleanor Ratcliffe, Francesco Aletta, Rossano Albatici, Francesco Babich, Tin Oberman, Jian Kang

**Affiliations:** ^1^Institute for Renewable Energy – Eurac Research, Bolzano, Italy; ^2^UCL Institute for Environmental Design and Engineering, The Bartlett, University College London, London, United Kingdom; ^3^School of Psychology, Faculty of Health and Medical Sciences, University of Surrey, Guildford, United Kingdom; ^4^Department of Civil, Environmental and Mechanical Engineering, University of Trento, Trento, Italy

**Keywords:** COVID-19, noise, indoor soundscape, acoustic comfort, wellbeing, WFH

## Abstract

The period of home confinement during the COVID-19 pandemic made the importance of a high-quality surrounding environment even more evident than before. Several studies have been carried out to assess the (negative) impacts of noise on annoyance, particularly whilst working from home (WFH). The present study takes a step further by (1) investigating the positive and negative impacts of the “actual” acoustic environment on a range of activities, i.e., WFH, relaxation, physical, and sexual activities, and (2) identifying the characteristics of an “ideal” indoor soundscape. The study is based on the qualitative analysis of verbal descriptions collected from open-ended questions included in a survey administered in January 2021 to 464 respondents living in London, during the COVID-19 lockdown. The range of impacts in the actual scenario varied from no effect on task execution, to disruption, distraction, concern of disturbing others or being heard. Positive impacts included support of concentration, relaxation, motivation, freedom of sound expression, feeling of being connected to the surroundings and comforted by the presence of others, according to mechanisms described in the study. Negative appraisal could trigger coping strategies (e.g., controlling windows, playing music, wearing headphones) and behavioural changes (e.g., lowering the volume of the voice or music, muting oneself during call, changing workout type) that could in turn limit or enhance the freedom of behaviour, affect or foster wellbeing. Negative impacts were most frequently reported on WFH (by 55% of the participants), followed by relaxation activities (40.6%), sexual activities (30.1%), and home workout (20.1%). The ideal soundscape was described as a quiet, well-sound insulated environment, which guarantees access to positive sounds (i.e., natural sounds, music, urban background), thus resulting in privacy, intimacy, and a place where to express themselves without noise-related constraints. The study complements literature findings on housing design directions in light of the COVID-19 pandemic, by providing further evidence on the impacts of poor sound insulation at home, the potential benefits of nature-based solutions for positive indoor soundscapes, and opportunities for an activity-based design of domestic environments, inclusive of a broader set of home uses and household compositions.

## Introduction

A growing body of literature is providing evidence on the impacts of the built environment on building occupants (European Environment Agency, 2020). This evidence is important to understand how to design and operate buildings in order to support and enhance people’s health and wellbeing (Altomonte et al., 2020; Awada et al., 2021a), where access to a healthy environment has been recently recognised by the United Nations as a human right (United Nations, 2022). It should be noticed that, in the context of research and practise on “healthy buildings,” the terms “health” and “wellbeing” are often paired, but their distinction appears quite blurred. Indeed, the “health” definition provided by the World Health Organization someway includes the “wellbeing” construct: “*health is a state of complete physical, mental and social well-being and not merely the absence of disease or infirmity*” (World Health Organization, 2020). Whilst an in-depth discussion on terminology can be found elsewhere (Hanc et al., 2019), we will refer in the following to “wellbeing” as a multidimensional construct including, amongst others, aspects related to eudemonic, social, mental, physical wellbeing, environmental quality, comfort and satisfaction, productivity, and cognitive performance.

The recent COVID-19 pandemic and the related shifts in working and living patterns further highlighted the role that housing quality plays in shaping building occupants’ wellbeing (Amerio et al., 2020; Awada et al., 2022). Stay-at-home mandates forced a range of activities (e.g., home working, schooling, sport activities) inside dwellings that were not designed to accommodate such functions. Researchers have been investigating the suitability of the acoustic environment at home for this changed scenario, with the aim of redefining the design requirements for the post-pandemic housing.

When the focus is on the impacts of the acoustic environment on humans, the “soundscape approach” aligns with the “healthy buildings” framework in moving from “avoidance of annoyance and disease” towards “adding value” in terms of improved wellbeing through the built environment (Torresin et al., 2020b). Compared to the traditional noise control approach, soundscape research not only considers aspects related to noise exposure, annoyance, and mitigation, but acknowledges the importance of sound as a meaningful component in our everyday life (Ratcliffe, 2019; Guastavino, 2021). Whilst historically rooted in the context of urban planning, researchers have been more recently looking at its potential application inside buildings, where people spend most of their time and where the impact of the acoustic environment can therefore be the greatest (Torresin et al., 2020a). Indoor soundscape research aims to characterise the “*indoor acoustic environment as perceived or experienced and/or understood by a person or people, in the context defined by the building*” [(Torresin, 2022), adapted from (International Organization for Standardization, 2014)], in order to design spaces that support the execution of activities and improve people’s wellbeing.

As a result of the measures taken with the health emergency, the COVID-19 lockdown represented an unprecedented scenario in terms of outdoor noise levels reduction and concentration of activities in the domestic space (You et al., 2022). Several surveys have addressed the impact of the acoustic environment on building occupants (Şentop Dümen and Şaher, 2020; Andargie et al., 2021; Bartalucci et al., 2021; Caniato et al., 2021; Dzhambov et al., 2021; Lee and Jeong, 2021; Puglisi et al., 2021; Aumond et al., 2022). The focus has been more often on noise annoyance, perceived loudness, salience of specific noise sources, general evaluation of the acoustic environment (i.e., sound quality and appropriateness) and perceived magnitude of (negative) impacts of noise exposure, sometimes comparing the ratings collected during lockdown to those prior to lockdown.

However, three main aspects should be highlighted. Firstly, the traditional focus on the negative impacts of noise exposure does not allow information to be drawn on potential positive effects of desired sounds to inform the design of positive soundscapes (Torresin et al., 2019a).

Secondly, assessments have mainly focused on the impacts on the WFH (working from home) activity or on a global assessment of the sound environment, without referring to a specific activity. However, it must be considered that the soundscape is strongly context-dependent (International Organization for Standardization, 2014), and assessments should take into account that the appropriateness and quality of a sound environment depends on the activity for which the assessment is made (Torresin et al., 2019a). Moreover, if WFH was the main novelty during the lockdown, it was not the only element of change. The closure of gyms, for instance, and the limitation of travel possibilities induced many to workout at home. Such activities were complemented by those traditionally carried out in the domestic environment, such as relaxation, sleep (Office for National Statistics, 2020), and sexual activities. Aside from specific literature on noise effects on sleep quality, however, research on the impacts of the acoustic environment on relaxation, home workout, and sexual activities is, to the best of the authors’ knowledge, very limited or even missing, despite potentially being fundamental layers of people’s wellbeing (Engineer et al., 2021).

Thirdly, methods adopted in previous investigations almost exclusively relied on closed questions (e.g., rating scales), which are convenient in terms of speed of data collection and analysis. These methods, however, constrain the participants’ answers within the topics and categories included by the researchers. When answers are prescribed by the experimenter, results can reflect “*the researcher’s interpretation and understanding of the relevant concepts, rather than words that the public would normally recognize and use to evaluate a soundscape or place*” (Payne and Guastavino, 2018, p. 2). For these reasons, soundscape studies and technical specifications include methods (e.g., interviews, open ended questions) that allow for gathering spontaneous descriptions by participants to be analysed by means of qualitative methods (International Organization for Standardization, 2018; International Organization for Standardization, 2019).

Taking London as a case study, the present work aims to characterise the indoor soundscape in residential buildings, thus contributing to fill the research gaps highlighted above and overcoming some of the limitations present in the existing literature. Verbal descriptions, followed by qualitative analysis, have been gathered from open-ended questions included in an online survey administered to 464 respondents living in the UK (London area) and working from home in January 2021 during the COVID-19 lockdown (Torresin et al., 2021). The questions posed to the participants investigated the negative and positive impacts of the acoustic environment and their *desiderata* with reference to the following activities: WFH, relaxation, home workout and sexual activities. The use of open-ended questions encourages participants to reflect on the topic being discussed and freely report opinions, without constraints provided by the researcher. The study departs from the traditional noise control literature, which focus on the “noise exposure” – “noise annoyance” binomial, to derive a deeper knowledge of the negative and positive impacts of the sound environment (“the actual soundscape”) and the features of an “ideal soundscape” (Guastavino, 2006), with reference to the different activities explored. The “actual soundscape” refers to the acoustic environment that occupants are exposed to in their real life, with its negative and positive impacts, whilst the “ideal soundscape” results from an imagination exercise in which participants are called to imagine an optimal, ideal scenario.

The objectives of the present study are:

1. To identify the variables within the acoustic environment and the mechanisms through which those variables exert their influence on home working, relaxation, home workout, and sexual activity, thus comparing the magnitude of the impacts of the acoustic environment on building occupants for the different activities under investigation.
2. To identify the features characterising the ideal soundscape for home working, relaxation, home workout, and sexual activity.

Findings will be useful to discuss and outline design and research directions for post-pandemic housing.

## Materials and methods

### Questionnaire

The analysis is based on verbal descriptions collected in an online survey administered to adult participants *via* Prolific participant pool (Peer et al., 2017; Palan and Schitter, 2018) on 18 and 19 January 2021, whilst London was in lockdown (Statutory Instruments, 2021). Potential participants were filtered through the following pre-selection criteria available on the platform: age (18–65 years), no self-reported hearing impairments, indication of London (United Kingdom) as area of residence and WFH during COVID-19 lockdown. After excluding 9 participants who failed an attention check included in the survey, 464 participants (181 males, 282 females, 1 other; mean age: 32.2 years; SD: 9.1 years) were considered for data analysis.

The survey was designed following a mixed-method approach (Johnson and Onwuegbuzie, 2004), in order to tackle the complex and multi-faceted nature of soundscapes from several perspectives, according to the principle of triangulation in use in behavioural and social sciences (International Organization for Standardization, 2019), by combining quantitative data from closed-ended questions with data from open-ended questions. It included five main sections covering (1) the WFH activity; (2) leisure activities performed at home; (3) housing characteristics; (4) urban context; and (5) person-related traits. The survey employed a circumplex model of affect (Russell, 1980; Posner and Russell, 2005) for indoor soundscape assessment (Torresin et al., 2020a), based on 8 perceptual attributes, corresponding to two main dimensions (“comfort” and “content”) and two secondary dimensions (“privacy-control” and “engagement”). Previous analyses on the extended dataset relied on this coordinate system to investigate differences in soundscape evaluation according to a specific activity (i.e., WFH and relaxation) (Torresin et al., 2021), to identify impacts of several acoustical, building, urban and person-related factors on building occupants’ wellbeing and on soundscape dimensions (Torresin et al., 2022). The survey ensured a broad coverage of the city of London, both in terms of geographical area, building type and ownership status. A detailed description of the study design, of the building stock covered by the survey, and a questionnaire excerpt can be found in (Torresin et al., 2021).

The present study focuses on the qualitative analysis of responses given by participants regarding the positive and negative impacts of the sound environment with reference to the following activities: WFH, relaxation (i.e., watching TV, reading, listening to music), home workout, and sexual activities. Open-ended questions are listed in Table 1, whilst raw answers are available as Supplementary material. In the case of the impact of the sound environment on sexual activities, given the sensitive nature of the topic, the answer to the question was kept optional. The research was approved *via* the UCL IEDE Ethics departmental low-risk procedure on November 26th, 2020.

**Table 1:** Excerpts of the open-ended questions included in the survey.

Actual indoor soundscape	In your view, how is the sound environment currently (positively and negatively) affecting your working activity from home? (e.g., heard noises and sounds, building characteristics, urban environment)
	In your view, how is the sound environment currently (positively and negatively) affecting your leisure activities at home? (e.g., heard noises and sounds, building characteristics, urban environment) Whilst watching TV, reading, listening to music: During your sport activities at home: In your sexual activities at home:
Ideal indoor soundscape	In your opinion, what should the sound environment be like to allow you to work at best from home? (e.g., heard noises and sounds, building characteristics, urban environment)
	In your opinion, what should the sound environment be like to allow you to relax at best whilst at home? (e.g., heard noises and sounds, building characteristics, urban environment) Whilst watching TV, reading, listening to music: During your sport activities at home: In your sexual activities at home:

### Data analysis

Verbal descriptions were analysed through thematic analysis (Braun and Clarke, 2006), “*a method for systematically identifying, organizing, and offering insight into patterns of meaning (themes) across a data set*” (Braun and Clarke, 2012, p. 57). Thematic analysis allows us to “*make sense of collective or shared meanings and experiences*” across the data set in relation to specific research questions (Braun and Clarke, 2012, p. 57). The formation of the codes and themes followed a combination of inductive (or data driven) and deductive (or theory-driven) approaches, as it builds on the description of the participants’ experiences, but also on the theoretical soundscape framework described in the ISO 12913-1 standard (International Organization for Standardization, 2014).

In relation to objective 1 of the study, and in particular the identification of the mechanisms that lead the acoustic environment to generate an impact on people, it was useful to condense themes, sub-themes and their interrelationships into thematic maps (Braun and Clarke, 2012) addressing each one of the investigated activities.

A dedicated coding was carried out, in which the answers to the questions on the “actual soundscape” (see Table 1) were assigned to an impact category (i.e., negative, neutral, positive), to an “N/A” category (in the case of people who state that they do not play sports at home or are not sexually active) or to an “other” category, in the case of irrelevant answers. The categorisation is based on a simplification of the message reported by respondents, which often includes complex evaluations and descriptions of different and contrasting impacts. For the sake of analysis, it should be noted that under the category “negative impacts” are both negative evaluations and evaluations that include a mix of negative and positive impacts. This is useful to provide an assessment of which activities are most negatively influenced by the surrounding sound environment at home, in terms of percentage of impacted people.

As regards objective 2, participants’ responses to open-ended questions on ideal soundscapes were analysed using the method of constant comparison of data (Hallberg, 2006). The analysis led to the identification and coding of elements which feature the ideal acoustic environment with reference to the different activities under investigation. The list of *desiderata* concerns both characteristics that should and should not be present in the ideal soundscape. Occurrences within each code were summed across the participants, and then analysed through descriptive statistics.

Coding was managed in NVivo software (QSR International). Codes with less than five occurrences have not been retained.

## Results

The following sections include the results of analyses leading to the description of the actual (objectives 1) and ideal indoor soundscapes (objective 2), with reference to the different activities under investigation.

### Actual soundscape

Four main themes were identified, underlying the impact of the surrounding sound environment on people. Themes concern (1) the context in which the activity takes place, (2) the characteristics of the acoustic environment, (3) the soundscape interpretation, and (4) the adoption of coping strategies by the building occupants. Sub-themes as well as their mutual relationships are interpreted with some differences according to the specificities of each activity, as described in the following paragraphs.

#### WFH

**Figure 1:**
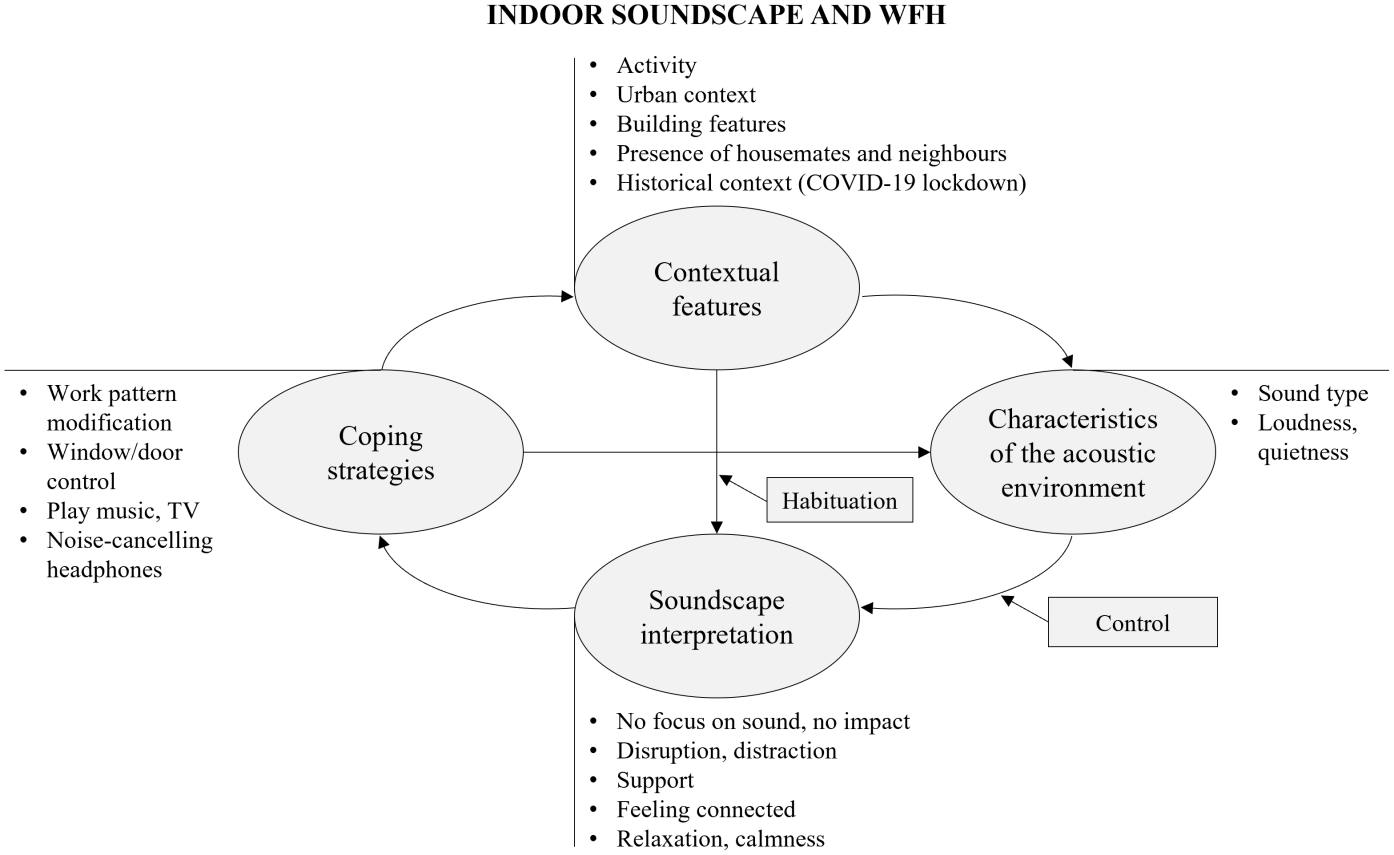
Thematic map on the relationship between indoor soundscape and WFH showing themes (within rounded boxes) and sub-themes, together with their causal interconnections and moderating factors (within squared boxes).

The first theme concerns the context in which the evaluation is made, and this includes the functional context (i.e., the activity being performed), as well as the historical (i.e., lockdown during the COVID-19 pandemic), situational (e.g., living with housemates or family, having neighbours), and the spatial ones (i.e., urban area and building features). WFH is related to the demand for concentration and intelligibility during calls through collaboration apps. The unprecedented historical context emerges in details referring to the specific lockdown condition, the related psychological state, and the sharing of domestic space with other cohabitants. Situational aspects include sharing the living space with others, such as housemates or family members, and being exposed to neighbours. The quality of the spatial context is defined by the urban area and building features, which include for instance the exposure to a busy urban area, the availability of a quite side, such as a courtyard, and good sound insulation of building elements (e.g., walls and windows). Contextual features contribute to shape the acoustic environment at home, as conceptually represented in the map in Figure 1. Exemplary excerpts for subthemes are reported in Table 2.

**Table 2:** Example of excerpts and coded fragments (underlined) related to themes and sub-themes defined from the thematic analysis for soundscape impacts on WFH.

Theme	Sub-theme	Excerpt
Contextual features	Activity	Sometimes when in online meetings, I cannot hear my colleagues as the traffic noises are too loud.
	Urban context	My bedroom which I use to work from home is situated at the back of the house, facing the big garden. I live on a busy road with lot of people walking past and talking, as well as hearing traffic and sirens, which distracts me from my work.
	Building features	It’s good that I do not hear street noise much at all as we are high enough up as well as having thick enough glazing.
	Presence of housemates and neighbours	Sometimes my roommate will spent a lot of time in the kitchen cooking, and she can be quite loud. Neighbours / other residents walking past my house can impact me negatively, the noises are irritating and distracting.
	Historical context (COVID-19 lockdown)	I like having some background noise and hearing the sounds for the car mechanic garage helps to remind me there is an outside world (especially in the current pandemic). The sirens remind me that there’s a pandemic on and do make me feel sad.
Characteristics of the acoustic environment	Sound type	The natural sounds from outside positively affect my working activity by providing a sense of calmness. Building work is particularly loud, as there is a near construction project and we have thin single glazed windows which is terrible when trying to speak and listen on calls, and carry out individual work.
	Loudness, quietness	My street is very quiet so it positively affects my working from home. Sometimes it can be “too quiet” and makes you lose focus feeling alone. The courtyard of our building overlooks a primary school playground so when students are attending that can be very loud during their breaks and lunchtimes.
Soundscape interpretation	No focus on sound, no impact	It’s not so bad. I would say it’s pretty ignorable. Not much affecting, when I work mostly focused on the thing that I am doing so I ignore all other things.
	Disruption, distraction	People or vehicles passing by outside talking will suddenly pull me out of focus. I have a really hard time dealing with noises coming from the rest of the house, especially when people have very loud conversations next door, as it’s quite disruptive.
	Support	The environment is positively conducive to me getting work done and there are no negatives that I cannot handle. I live in a residential area so hear very minimal noise which is good as I can focus better and not be interrupted during online meetings Positively, background noise like wind, cars, conversation helps me to focus a lot more.
	Feeling connected	The little noises I do hear can make me feel less lonely. It’s a bit noisy outside but I like feeling connected to the outside world even if it’s just hearing the traffic!
	Relaxing, calming	I live in a relatively quiet residential area which is quite calming. I get to hear birds and once, a squirrel going down the roof, which did distract me, but it made me happier. Living close to a park, I believe that the sound of nature (e.g., birds, etc.) can help relax and focus.
Coping strategies	Work pattern modification	I need to start work earlier to avoid the noise from the opposite school. When I have zoom meeting the background noise is not ideal so this is negative factor but I am easily able to move to a different room in the house which does not get the noise. I constantly have to mute myself so the noise does not affect others.
	Window/door control	Any time I have a meeting I close all my windows so noise will interrupt my meeting
	Play music, TV	I usually listen to music or tv which I would not do at work so that cancels out most of the outside noise When I work by myself, I need to turn the music up in my headphones so that I can focus on that rather than the noise coming from the outside.
	Noise-cancelling headphones	I’ve to use noise cancelling headphones.

“The inability to control, or be in control, of most urban noises and the sounds of others also working in isolation drives me insane.”

“Sometimes I have to listen to music in order to block out the noise coming from neighbors but listening to music negatively affects my concentration. I still choose music because at least that’s something I can control.”

Quietness availability and the lack of intrusive noise are key for a sound environment that allows concentration and is conducive to work. If living in noisy and loud environments can be highly disruptive, silent environments are sometimes evaluated as rather bland and not supportive. Natural sounds are generally described as calming and relaxing. Music can enhance concentration and serve as sound masking. Listening to family and urban sounds in the background can be helpful compared to a completely silent environment, as it could alleviate the feeling of loneliness, help one feel connected to the outside world and comforted by the presence of other people, particularly during the COVID-19 emergency.

It must be noticed that the sound environment does not always have an impact on work. Some respondents were able to “block out the noise” and isolate themselves by concentrating on the activity they were engaged with. In other cases, habituation was identified as a factor moderating the association between context and sound interpretation, as shown in Figure 1. Some of the respondents stated that they had become accustomed to their sound environment over time, indicating habituation to the acoustic conditions they were exposed to.

“The background noise – traffic etc (I live on a busy road less than a km from a hospital) is something I am used to and very rarely impacts me.”

When the interpretation of the soundscape leads to a perception of disturbance and lack of concentration, this can prompt behavioural actions. In addition to closing doors and windows, the main coping strategies include the use of noise-cancelling headphones, listening to music or podcasts, turning on the TV and adjusting its volume in order to mask unwanted noise, saturate the environment with preferred sounds and, in this way, promote concentration. However, TV, podcasts and music listened to by the respondents could also be sources of self-distraction in some cases. If control actions are not feasible or ineffective, adverse sound conditions lead to a behavioural change in the working activity, such as being on mute to prevent noise from intruding into the call, moving to a different place within the house or working during quieter times.

Behavioural actions thus may change the context (e.g., working patterns) and the acoustic environment (e.g., background noise level), as conceptually depicted in Figure 1.

#### Relaxation

A specific question investigated the impact of acoustic conditions on leisure activities, grouping the impacts on listening to music, watching TV, and reading. The functional context, described by the type of activity, was relevant in determining soundscape impacts. The activities included in the question resulted to be differently impacted by the sound environment, with a substantial difference between concentration activities carried out mostly in silence, such as reading, and activities that instead involve generating and listening to sounds or spoken messages, such as listening to music and watching TV. Other contextual aspects relate to the characteristics of the urban area, the construction quality of the home, and the presence of housemates and neighbours, which influence the acoustic environment in terms of sounds heard and available sound insulation. Finally, another factor often mentioned is the time of day at which relaxation activities are carried out. It should be noticed that details about the historical moment (i.e., the COVID-19 pandemic) are gradually omitted. In compiling the answers to subsequent questions, only aspects that are peculiar to the activity under consideration were further reported by the respondents. Exemplary excerpts for subthemes are reported in Table 3.

**Table 3:** Example of excerpts and coded fragments (underlined) related to themes and sub-themes defined from the thematic analysis for soundscape impacts on relaxation.

Theme	Sub-theme	Excerpt
Contextual features	Activity	Sometimes the traffic noise can dominate if there are no other sounds, which is negative. Mostly if I am watching TV, there are no negatives. If I were reading, it can be distracting. When reading however the noise of pets, neighbours etc. can be very distracting and put me off.
	Urban context	Whilst the second living room is on the front of the house, which means the road is close by, I do not hear a lot of cars or traffic as my road is 90% of the time very quiet. This means that when I am in the living room I can totally relax and not be stressed out by noises from outside. Living by a canal means that often people on bikes or exercising play very loud music which can sometimes overpower what I am listening to and disturb me.
	Building features	It is fine as again I have double glazing windows and it keeps a lot of sound out.
	Presence of housemates and neighbours	I usually like to hear my children playing. I can quite often hear the neighbour’s children playing or crying which drives me mad.
	Time of the day	By the time I have finished work and watching tv etc. it is quite late and the construction has finished for the day.
Characteristics of the acoustic environment	Sound type	The flat overlooks a parking lot, with too much car noise from residents, deliveries etc. Our dog can also be fairly loud as can other dogs in the neighbourhood and if one starts barking they all tend to join in.
	Loudness, quietness	Noisiness from building makes it hard to relax, cannot hear tv series. But when there are no building noises, the environment is fine for leisure activities. I think my environment is good for relaxing and reading or watching TV as my living room is quiet, and so activities are rarely interrupted.
Soundscape interpretation	No focus on sound, no impact	The sound can sometimes be loud, however, it does not affect my leisure activities. In terms of doing a leisurely activity there is less focus on the sound environment as you do not need a high level of concentration to do this. These are often done with headphones, so it limits the level that sound environment had an impact.
	Disruption, distraction	When I’m reading I cannot focus because of the noises. I can hear people’s television and conversations in the evening, and this can be more disruptive. It is the case that it has been so loud, I could not hear what I was watching at a comfortable level.
	Worried to disturb others	Whilst watching TV, I have to be aware of the volumes as you can hear from other rooms. I cannot have it so loud that it disturbs the neighbours because the walls are thin, so if I want to listen to music really loud I have to wear headphones.
	Feeling connected	When the windows are open hearing the sounds of birds and people chatting is nice as you feel less isolated. I like hearing outside noises to keep me feeling connected.
	Relaxation	Nice to have people around, it feels homely and relaxing.
Coping strategies	Play music, TV	The sounds present are quiet enough that I can just put on my headphones and block most of the sounds out by whatever is playing through my headphones. Watching TV and playing music usually cancels out all external sounds if I set the volume high enough (not too loud to damage hearing, but not too low either). I put music on if I want to ignore it.
	Adjust volume	Have to increase the volume of the gadget sometimes to hear better because of the traffic noise. I tent to turn the volume up on the TV so that I do not get distracted by the noise from outside
	Window control	If it is too noisy outside I will close the window, so as to not hear the sounds outside

The second theme is related to the characteristics of the acoustic environment, notably sound type and sound dominance. The sound sources most often mentioned are family members or housemates, natural sounds, traffic, neighbours, construction works, TV sounds, music and podcasts played whilst WFH, people outside, and, to a lesser extent, pets, building services, home deliveries, and noise from gardening activities. Besides the type of sound source, the degree of quietness or loudness in the environment is a key piece of information reported by respondents.

The contextual and acoustic features contribute to determine soundscape interpretation, identified as the third theme by the thematic analysis. The sound environment was often reported as distracting and disruptive to work, as in the case of intrusive noise from people at home or outside, neighbours, loud traffic noise coming from the outside, or construction works. The perceived control over sound sources can be identified as a factor moderating the relationship between the exposure to the sound environment and its interpretation, as conceptually depicted in Figure 1. Notably, the lack of control over urban sounds and noise generated by neighbours can lead to annoyance and frustration, whilst controlling the sound environment with music or noise-cancelling headphones leads to positive evaluations.

**Figure 2:**
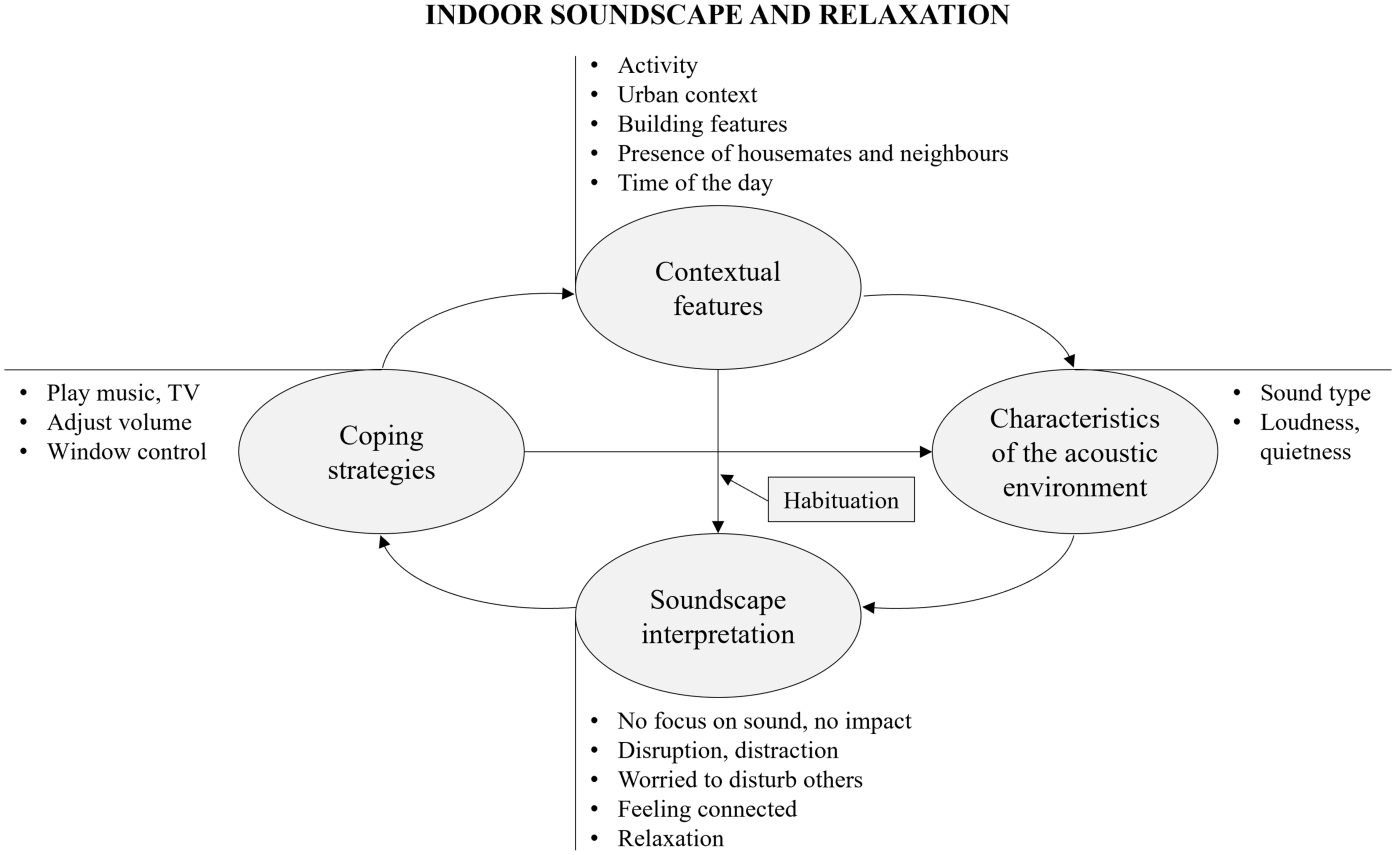
Thematic map on the relationship between indoor soundscape and relaxation showing themes (within rounded boxes) and sub-themes, together with their causal interconnections and moderating factors (within squared boxes).

The context contributes to determining the acoustic environment, as conceptually indicated in Figure 2. Leisure activities often occur in the evenings, when road traffic is reduced and construction works are over, resulting in a quieter environment. Mentioned sound sources were neighbours, traffic noise, other people living at home, natural sounds, TV and music played by the respondents, construction works, people outside, and, to a less extent, pets, home appliances, building services, and deliveries. Another aspect concerning the acoustic environment, as mentioned in the survey, is the dominance of such sound sources, or, in general, the degree of quietness or loudness in the environment.

Contextual and acoustic features contribute to determine soundscape interpretation. Sound stimuli can hinder leisure activities at home. This is especially true for reading, as noise can intrude, distract, and be off-putting. Noise, especially from neighbours and outside traffic, can also impair listening quality of music and reduce speech intelligibility whilst watching TV. However, these activities are generally less impacted by the acoustic environment compared to reading, as TV and music are often listened to with headphones or through loudspeakers, allowing to overpower the sound environment and shape it to according to individual’s preferences. Whilst quietness is a key element for reading, music and nature provide sounds that are conducive to relaxation. Moreover, acoustic stimuli can be beneficial by providing a connection to the outside word, and a feeling of being comforted and less alone. Loudness is often desired when listening to music, although respondents often reported a fear of disturbing other people in the house or neighbours, and, therefore, a limitation in the enjoyment.

No impact by the acoustic environment is finally reported by many. This is because leisure activities are often carried out at quieter times, such as evenings or weekends. Moreover, music and TV can provide useful masking opportunities against the present noise. Many are able to ignore the sound environment whilst being focused and engaged on the activity. Finally, habituation was found to be a factor that moderates the influence of the urban context on sound interpretation, leading to a lack of reported impact.

“The positive side of sound where I live is fine as I have lived in this type of environment most of my life.”

“It doesn’t affect it too much as I've learnt to live with the noise.”

Under conditions of dissatisfaction, viable coping strategies are turning up the volume of the TV or music because of a noisy environment, turning down the volume or wearing headphones so as not to disturb others, and closing windows to block out external noise. Behaviour can in turn alter activity and re-shape the acoustic environment, as indicated in Figure 2.

#### Home workout

When asked to reflect on the impacts of the acoustic environment on sport activities at home, 28.4% of the participants reported they did not play sport or did not do it at home (N: 132). Relevant answers were collected by 68.7% of the respondents (N: 319; “others and irrelevant” 2.9%, N: 13). Amongst those exercising at home, the type of physical activity that is carried out and the time of day at which it is performed were relevant aspects in defining the impacts of the sound environment. Sports activities at home are often practised with the aid of headphones and video devices from which to follow instructors and training programmes. As it will be highlighted in the following, a differentiation of impacts occurs for activities such as yoga and meditation, which require concentration and a sound environment conducive to this. As per leisure activities, home workout is often performed at non-working hours, such as in the evening, and early morning. Finally, the urban context, the housing quality (e.g., new windows and sound insulated dwellings), the presence of family, housemates and neighbours contribute to frame the context and determine the sound environment in which home exercise takes place. Exemplary excerpts for subthemes are reported in Table 4.

**Table 4:** Example of excerpts and coded fragments (underlined) related to themes and sub-themes defined from the thematic analysis for soundscape impacts on home workout.

Theme	Sub-theme	Excerpt
Contextual features	Activity	Does not make a difference unless I am trying to do yoga, in which case I do not like hearing other noise made by people. For more high-energy activities such as stationary bike, or aerobics it is less of a problem as I usually play music. If the programme I am following has music accompanying the videos I tend to switch this off so I can hear the instructor better, and then play my own music more quietly.
	Urban context	The noise outside can sometimes distract me, so I tend to have to listen to a podcast in order to block this noise out. Noise can include building works and people on the street.
	Presence of housemates and neighbours	Negatively – cannot drown out family asking for things. If the neighbour decides to start hoovering next door it can make it difficult to hear the music whilst I’m working out.
	Time of the day	Often I’m dong pilates in the evening, when there is little noise happening.
Characteristics of the acoustic environment	Sound type	I usually play my own music when doing sport at home. The sound of cars and traffic sometime distracts me, and I often feel the urge to look outside when I hear sirens.
	Loudness, quietness	I feel lucky to live is such a quiet area. I do not want it to be too quiet as this is not conducive to working out. No external sounds would be a problem.
Soundscape interpretation	No focus on sound, no impact	It does not affect me. I do not mind about other noises as I’ll have my headphones on. To be honest not a lot - as I would be concentrating on the workout on a screen with music on in the background (either headphones or speakers) so would zone out of any noises!
	Disruption, distraction	I will however hear sounds from the TV if anyone else is home which can negatively affect my concentration on the workout.
	Motivation	I listen to loud music when I exercise or ambient music during yoga. The music I choose affects me positively; gets me in the mood and motivates me. It does not affect it negatively, in fact it motivates me in a way, you know. If it was pure silence, it would be hard to exercise without music or sound that can be music in a way. This is why sound would be important.
	Worried to disturb others	I do have to be mindful with how much noise I make. The only issue is that since I live in a flat, if I jump or do workouts that require a lot of leg work, it can disturb the downstairs neighbour.
	Relaxation	Nice and private to work out in so I can relax and do not feel uncomfortable. If I do exercise at home it’s all quiet and relaxing.
Coping strategies	Play music	I put music on if I want to ignore it. I play music when doing exercise which blocks a lot of the background noise.
	Adapt the type of activity	However, as the floors are also very thin, we have gotten a complaint before from our neighbours that our jumping (doing high intensity workouts) is quite disturbing, which means that we have to limit the number of jumping exercises we do every week or schedule it at other hours (not at evening time). This has negatively affected my exercise. At first I chose YouTube workouts that were specifically for people that live in flats and have neighbours downstairs. The exercises basically do not involve any jumping or heavy landing so that the noise is not causing a nuisance to your neighbours.
	With headphones	I use headphones when I workout so as not to disturb my neighbours as I like to have my music on loud. I wear headphones all the time during sporting activities so I am in complete control of the sound environment. In terms of the impact of the sound environment which I control, this is entirely positive insofar as it blocks out all other noise from the surrounding environment.

**Figure 3:**
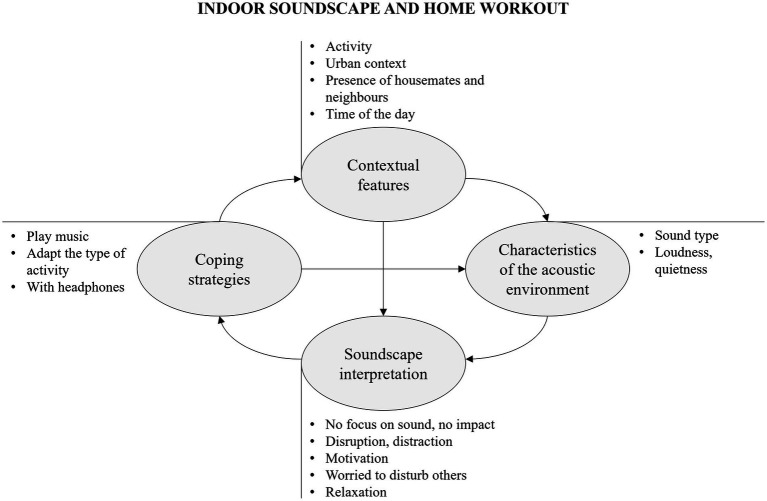
Thematic map on the relationship between indoor soundscape and home workout showing themes (within rounded boxes) and sub-themes, together with their causal interconnections.

As regards the second theme, sound type and quietness magnitude define the characteristics of the acoustic environment (see Figure 3). Music is the component most often mentioned amongst the sound stimuli during home exercise. Other sound sources are the sounds of nature, traffic, people living at home, and neighbours.

The impact of the sound environment on training at home is generally not relevant. Home workout is mostly accompanied by the reproduction of music, often through headphones, which can provide rhythm and motivation in the execution of the activity, offer opportunities for masking against ambient sounds and improve the intelligibility of instructions from online trainers. In addition, many respondents report that they do not pay attention to the acoustic environment whilst focusing on the sport activity. Sport activities are often performed in the early morning and in the evening, thus at quieter times. However, the presence of sounds and noises in general is rated as less critical, and indeed is desired to provide support and sound context in the performance of the activity. This is valid for music and natural sounds, but sometimes also for urban sounds or sounds related to the presence of someone at home. A certain degree of quietness is, however, required for activities that require concentration, such as yoga and meditation. In general, natural sounds, music and a quiet environment can help relaxation during home training, whilst noise from traffic, people at home or neighbours can be very distracting and impair concentration. Since sport activity often involves jumping and making movements that impact on the floor and playing loud music, this comes with the worry of disturbing others, such as neighbours and housemates.

Coping strategies include playing music to shape the sound environment to one’s liking, by masking present noises. In the case of airborne or impact noises that could be heard by others in the building, actions involve changing the type of sport activity, foregoing exercises that involve jumping, limiting the intensity of the exercise, lowering the volume of the music played by loudspeakers or wearing headphones whilst exercising.

#### Sexual activities

With reference to the optional question on the impact of acoustic conditions on sexual activities, relevant answers were collected from 51.5% of the respondents (N: 239, “others and irrelevant” 22.9%, N: 106), whilst 25.6% of the participants skipped the question (N: 119).

Contextual aspects included the activity (i.e., sexual activity), the presence of neighbours and housemates, the quality of the urban area and housing (i.e., house size, construction quality), and the time of day when sexual activity takes place. Good building construction was related to adequate sound insulation of the building elements. Since sexual activity often takes place in the evening or at night, these are the times when there is less traffic, and construction sites are not in operation, resulting in a quieter outdoor acoustic environment. Exemplary excerpts for subthemes are reported in Table 5.

**Table 5:** Example of excerpts and coded fragments (underlined in the table) related to themes and sub-themes defined from the thematic analysis for soundscape impacts on sexual well-being.

Theme	Sub-theme	Excerpt
Contextual features	Activity	Overall I would say the sound environment is positive for sexual activity whether with my partner or alone. Because of the lockdown, my flatmate and I are spending most of the time in the flat, which means that the sexual activities need to be sound-constrained.
	Urban context	Intrusive noises from the urban environment and heard noises such as voices negatively effect sexual activity at home. It is quiet, however I can sometime hear people in the courtyard outside which can be distracting
	Building features	Noise is not an issue as walls have insulation and can keep sound from travelling. Because I live in a big house this is not a problem.
	Presence of housemates and neighbours	The annoying thing is when you suddenly hear the neighbors walking up and down the stairs, distracting you a bit. Negatively impacted by hearing family in house - distracting, self-conscious.
	Time of the day	It does not affect it as much. Usually due to the time we have sex, it is quieter so less or no noise from neighbours and outside.
Characteristics of the acoustic environment	Sound type	We can hear every single cough, sneeze and snore that my elderly neighbour makes in her bedroom. That is alongside every conversation on the phone and tv, as well as when she turns on the light. It’s quite a passion killer! Sometimes distracting noise from others in the house. Birds chirping from time to time and river are having positive impact.
	Loudness, quietness	Positive sound environment. It is quiet and not much noise so we don't get disturbed. I prefer louder outside noises during these times.
	Temporal pattern	If there is an abnormal pattern of noise then I feel anxious. Quiet natural sounds without sudden noises would be good
Soundscape interpretation	No focus on sound, no impact	Don’t pay attention to external factors when engaging in sex. The noise has no impact on mine and my partners sexual activities.
	Privacy concern	The bedroom is quite close to the corridor so I am sometimes concerned with neighbours hearing this. It makes me wonder whether me and my partner can be heard by neighbours and others in the house as I am aware of the sounds I am able to hear on a day to day basis. fear of being overheard by children is always at the back of the mind. I do worry that my housemates will be able to hear me and my partner which can be frustrating
	Disruption, distraction	So sounds of other people will generally be more distracting to me during sex than they might be at other times. Disruptive and uncomfortable, often in need of more peace to start sexual activities.
	Positive emotional impact	I feel with freedom and not interrupted or feeling that I am disturbing anyone.
Coping strategies	Sexual behavioural change	I can often hear people outside my bedroom window which can make me feel self-conscious during sexual activities and sometimes make me decide to stop. The awareness of sound spillage between the flat does affect negatively the freedom to engage in sexual activities without having to worry about being quiet.
	Window/door control	I would keep windows closed because otherwise you can hear neighbors and they can maybe hear you.
	Play music	I have control over my sound environment and can play music, put on TV, etc.

The characteristics of the acoustic environment mainly concern the type of sound, sound dominance and temporal patterns. The most often mentioned sounds are those generated by other people, whether neighbours, family members or housemates, and the music played in background during the sexual activity. To a lesser extent, traffic noise, sounds of nature or sounds generated by the sexual activity itself are reported. Other aspects are the degree of quietness or loudness of the acoustic environment and the temporal characteristics of the sound phenomenon, such as the presence of sudden or repetitive noise.

**Figure 4:**
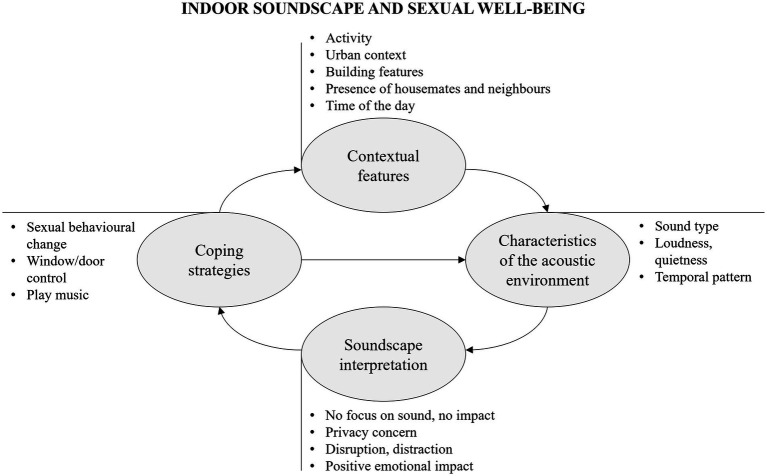
Thematic map on the relationship between indoor soundscape and home workout showing themes (within rounded boxes) and sub-themes, together with their causal interconnections.

Contextual and acoustic features drive soundscape interpretation, as depicted in Figure 4. For many, the acoustic environment is not influential during sexual intercourse. The focus on the activity allows many to get away from the surrounding environment and to “block out” external sounds. For some, this is justified by the availability of a quieter environment free of disturbing noise (e.g., traffic or construction works) when this activity takes place. Amongst those reporting impacts of the acoustic environment, sounds other than those generated by sexual activity can disturb, distract and interrupt the sexual activity itself, such as in the presence of voices or sudden noises. Sounds associated to the presence of people (children at home, neighbours, housemates) generate embarrassment, frustration and concern for privacy, as hearing someone is felt as synonymous with being heard. On the contrary, the availability of a quiet and adequately sound insulated environment, the presence of positively perceived sounds (e.g., natural sounds and music) can help to shape a private and intimate context in which to experience sexual activity. This would provide a positive impact on emotion and behaviour, allowing to focus on the sexual intercourse, without concerns or distractions, and with freedom of expression.

When soundscape interpretation leads to the perception of disturbance and lack of privacy, this triggers behaviour. Coping strategies include opportunities to control the acoustic environments, such as closing doors and windows, playing music, or turning on TV. Playing music helps to tailor the atmosphere with sounds of preference, whilst also covering up any unwanted noise that may be present. Moreover, music and TV sounds help to provide masking opportunities against the sounds generated by sexual activity, thus improving privacy. If control actions are not feasible or not sufficient, adverse acoustic conditions lead to a behavioural modification of sexual activity, such as adapting and limiting sexual intercourse in order to be quieter, or even stopping it. Behavioural actions thus may change the context (e.g., the sexual activity itself) and the acoustic environment (e.g., background noise level), as conceptually depicted in Figure 4.

#### Comparison of indoor soundscape’ impact on different activities

**Figure 5:**
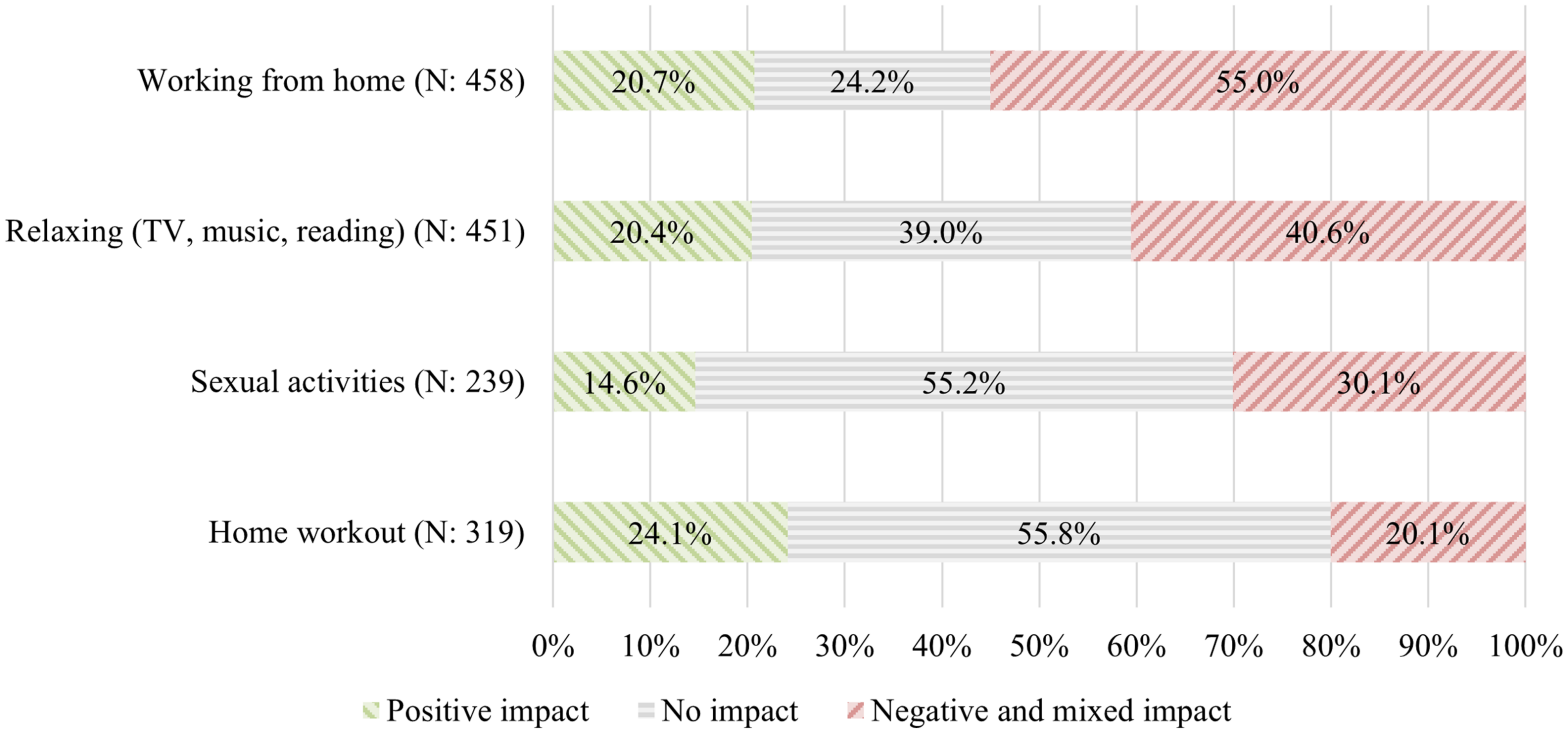
Percentage of respondents who reported a positive impact, no impact, and negative or mixed impacts of the noise environment on WFH, relaxation, sexual activities, and home workout.

Figure 5 shows the percentages of participants who reported an (exclusively) positive impact, no impact, or negative (or mixed) impacts of the sound environment on the different activities investigated. The acoustic environment negatively affects WFH for 55.0% of the respondents. Working at home is the most impacted activity, followed by relaxing activities (40.6% of participants reporting negative impacts), sexual activities (30.1%), and working out at home (20.1%). The percentage of respondents reporting positive indoor soundscapes is 20.4–24.1% for home working, relaxation, and physical activity. Positive impacts are lower for sexual activity, where only 14.6% of participants describe positive soundscapes. Regarding the effect on physical exercise and sexual activities, it should be noted that for most people (around 55% of participants) the acoustic environment has no effect.

### Ideal soundscape

The following sessions describe the features of indoor soundscapes that are ideal for performing different activities at home. Percentage of respondents who reported a desire for the presence, increase, decrease or absence of a certain feature in the ideal soundscape are provided in Table 6 for the activities under investigation.

**Table 6:** Percentage of respondents who reported the willingness of presence, increase (+), or decrease (−) of a certain feature in the ideal soundscape for the different activities investigated.

	Relaxing (N: 426)	WFH (N: 449)	Sexual activities (N: 204)	Home workout (N: 296)
+ Outdoor sounds (generic)	1.6%	2.9%	0.0%	2.0%
- Outdoor sounds (generic), + sound insulation from external noise	−16.4%	−12.0%	−8.3%	−6.8%
+ Traffic	2.8%	5.3%	5.4%	2.7%
- Traffic	−13.6%	−22.9%	−3.9%	−6.1%
- Construction	−6.1%	−9.1%	−1.0%	−1.7%
- Indoor sounds (generic)	−1.2%	0.0%	0.0%	−0.7%
- Appliances	−1.6%	−1.6%	−0.5%	−0.7%
+ Building services	0.5%	1.3%	0.0%	0.0%
- Building services	−1.2%	0.0%	0.0%	−0.3%
+ Human sounds (generic)	1.6%	4.7%	0.0%	0.7%
- Human sounds (generic)	−2.8%	−7.6%	−11.3%	−3.0%
+ Human sounds from outside	1.6%	1.8%	0.0%	0.0%
- Human sounds from outside	−3.8%	−2.9%	−0.5%	−0.3%
+ Human sounds at home	2.3%	3.6%	0.0%	0.7%
- Human sounds at home, + sound insulation between rooms	−7.0%	−14.5%	−3.4%	−3.7%
- Neighbours, detached house, + sound insulation between flats	−16.2%	−13.8%	−15.2%	−7.1%
+ Music, TV	*	17.6%	17.2%	47.0%
- Music, TV	*	−1.1%	−1.0%	0.0%
+ Natural sounds	26.8%	44.1%	9.8%	9.8%
- Natural sounds	−3.5%	−2.7%	−2.0%	−2.0%
Quietness	64.1%	55.9%	46.1%	30.7%
With some sound	11.3%	19.4%	12.3%	12.8%
+ Sound insulation (generic)	3.8%	3.1%	15.2%	2.7%
+ Sound insulation not to disturb others, free to make noise	3.8%	2.0%	28.4%	11.8%
Constant, no intermittent noise	2.1%	2.0%	0.0%	0.0%
- Sudden, unpredictable noise	−1.6%	−1.3%	−2.5%	−0.7%
+ Control over the environment	5.2%	7.8%	3.9%	2.0%
With headphones	1.4%	2.2%	0.0%	9.5%

Percentages are calculated on the number of participants who addressed aspects relevant to the design of the indoor soundscape. *Not considered as part of the activity itself (i.e., listening to music).

#### WFH

The ideal soundscape for home working is free of loud, intrusive noises, such as those from outside traffic, emergency vehicle sirens, people at home or neighbours, and construction sites. The sound environment is not distracting and promotes concentration. Quietness is the feature most often mentioned (see Table 6), allowing calls without interruptions or the need to mute. Higher sound insulation is desired, whether between adjacent rooms in the home, with neighbours or from outside noise. However, the ideal sound environment, for many, is not completely silent and the possibility of control over the sound environment is a key component:

“It should be controllable - as sometimes I need background noise and sometimes I don’t.”

The sounds of nature are most desirable and promote a calm and peaceful indoor soundscape. Music enables shaping of the sound environment according to individual preferences, whilst urban noises, such as a background of road traffic and voices, can help one feel connected and more productive. Window opening and music playing (often through headphones) are invoked as actions that can customise the acoustic environment, making it flexible in terms of desired loudness. This is captured by a participant stating:

“Quiet but not silent – I will open a window partly for fresh air but also to feel that there is some kind of human existence out there, it creates a feeling of being uninterrupted but also not alone. I like to hear the sound of the occasional car go past, or somebody speaking in a foreign language or the birds. I do also however like to play music to help me focus, not too loud and not too quiet though.”

In some cases, a background hum similar to that in the office is desired. For many, this supportive background must be constant, without sudden sounds.

#### Relaxation

The ideal soundscape during the investigated relaxation activities (i.e., watching TV, listening to music, reading) is mostly quiet (see Table 6). This is especially important during reading activities to allow immersion, and concentration without distractions. The absence of loud noises is important also whilst watching TV, to allow proper intelligibility without the need to turn up the volume to uncomfortable levels. Noises from outside, traffic or neighbours can be particularly intrusive, as well as from people at home. As already pointed out, the demand for quiet is often not synonymous with a sound-free environment, as rendered in the following excerpt:

“Quiet but not devoid of sound. Some background noises (like humming) can be soothing and help relax when reading.”

The ideal soundscape is relaxing and peaceful. Besides music, which is an underlying sound in the activity under investigation, sounds of nature are favoured, but a background of human sounds or the distant hustle and bustle of the city can also be beneficial. Sound insulation in facades and interior partitions and with neighbouring housing units is crucial. This reduces the amount of intruding sound and makes it possible to listen to music or TV at the desired volume without worrying about disturbing others or having to wear headphones.

“I would like to know that I'm able to be as loud as possible, when listening to music or watching TV, if I wish so, and that would not disturb anyone, neither at home, nor the neighbours. I wish the same would apply to the sounds penetrating into my house. I don’t want to hear the sounds neither from the other rooms in the house, nor from the neighbours. I would love to hear more birds singing, though.”

The ability to have control over the acoustic environment is crucial, allowing the acoustic environment to be tuned as desired: from a quieter environment when focus is required to a louder setting to be comforted and supported with the sounds of the city or of people at home.

“While watching tv or reading, I enjoy low level hubbub noises of everyday life. I find it comforting to hear other people in my life going about their business. While I would not appreciate loud disturbing noises, I find silence unsettling.”

“I like the atmosphere of my street and neighbourhood. I enjoy the little reminders of the world outside.”

#### Home workout

During sports exercising at home, the demand for quietness is lower than during other activities (see Table 6). The sound environment must be quiet enough to allow listening to the instructor’s instructions in online training programmes at a comfortable volume and to allow optimal concentration during yoga and meditation sessions.

“I think ideally for something like yoga, it would be very quiet or minimal white noise. Music or self-controlled sound is best for a workout. I think that it is better that it is not entirely silent. I think some noise helps to mask some sounds generated when you strain your body, and keep you motivated.”

“The sound environment which would be ideal for sports activities would be a fairly quiet one. I usually practice yoga and loud noises interrupt the meditative flow.”

Disruptive noises are, in particular, those of neighbours and those from outside, such as traffic. Music is an essential component in accompanying training sessions, as it can motivate, set the pace and saturate the environment with sounds of one’s liking.

“Less concentration is required, but personally I enjoy background music. Something with a solid beat that allows you to move in time.”

“With a lot of music, to keep up your fitness and encourage you!”

Natural sounds are also welcome. Good sound insulation is especially important in relation to other housing units, so as not to disturb neighbours with loud music or impact noise on the floor generated during training.

“It would be good to have sturdy floors so you’re not worried that you yourself are creating disruption by jumping up and down and making the house creak.”

“I would like to be able to do my workout and have some music on without disturbing others. This is pretty hard to achieve in London homes these days, the walls are literally cardboard.”

#### Sexual activities

The ideal soundscape for sexual wellbeing at home requires privacy and the ability to express oneself freely, without noise-related constraints. This results in a demand for greater sound insulation, especially from neighbours (see Table 6).

“The home should be built to absorb sound and ensure that no ambient noise ‘bleeds’ into other people’s homes and causes an unwelcome distraction.”

The environment should be quiet and free of intrusive noise, “*except for noise that I or my partner make/cause*,” as stated by one participant. Human-made sounds are particularly disruptive, as hearing other people is synonymous with concern of being heard, thus affecting perceived privacy.

“In particular, I would not want to hear the sounds of neighbours as it would imply that they can also hear you. This would be inhibitive for sexual activity as the sounds made are quite telling of what you are doing.”

“It would be best to not be disturbed by conversations, hearing other people or loud noises in other rooms as it ruins a private moment by reminding you people are around.”

Background noise, however, is often welcome, as it covers the noise generated during sexual intercourse. Natural sounds are beneficial, and music is employed to shape the atmosphere and to provide masking opportunities.

“Music played aloud in room for further privacy.”

## Discussion

### Impacts on domestic activities by the actual indoor soundscape

The impact of the acoustic environment on domestic activities is described by four themes derived by the thematic analysis of occupants’ lived experiences during the COVID-19 lockdown in London.

#### Contextual features

The first theme relates to the context in which the assessment of the acoustic environment is conducted, focal aspect in soundscape definition (International Organization for Standardization, 2014). The context is first of all functional, as each activity differently drives expectations and needs. The impact of the acoustic environment depends on the degree of interference with the task (Zimmer et al., 2008), and the ability to meet the requirements. The context is then defined by urban and building characteristics. The quality of the urban environment includes aspects such as proximity to transport infrastructures and green areas or the availability of quiet sides such as inner courtyards. The construction quality of the building is often mentioned in relation to the sound insulation performance of façades, partition walls, floors and windows. Situational factors include sharing space with family, housemates or exposure to neighbours. Part of the contextual framework is the historical period in which the study was carried out. Although not always explicitly mentioned, the working and living conditions at home were determined by the pandemic, which, due to its extraordinary and emergency nature, had an impact on people’s psychological conditions, as widely reported in the literature (Tull et al., 2020).

#### Characteristics of the acoustic environment

Urban context, situational and building features contribute to determine the surrounding sound environment, the second theme derived from the thematic analysis. The mentioned sound sources are those located in the outdoor environment, such as road traffic and, to a lesser extent, rail and air traffic, emergency vehicle sirens, construction sites, people in the street, natural sounds generated by living organisms (*biophony*) and by rain, wind, etc. (*geophony*) (Pijanowski et al., 2011), neighbours, and sounds generated indoors by other people, pets, building services and appliances. Interestingly, amongst the mentioned noise sources that are representative of the COVID-19 lockdown are home deliveries, which increasingly became an essential service during the period of confinement (Figliozzi and Unnikrishnan, 2021) and resulted in the emergence of an associated noise (i.e., ringing of doorbells at home or at neighbours). In addition to the type of source, the sound environment is described by the degree of quietness and noisiness, an aspect which will be discussed more extensively in the following sessions. Finally, the temporal pattern is described, with a distinction between constant sounds and impulsive and sudden noises.

#### Soundscape interpretation

The third theme concerned soundscape interpretation. Interestingly, the study allowed to draw insights into soundscape appraisal not only in relation to restoration from stress in relaxation activities carried out at home, but also with reference to new demands that emerged during the pandemic (i.e., WFH and home workout) and to activities traditionally carried out at home but whose link with sound stimuli had not yet been investigated (i.e., sexual activities). This activity-based perspective is crucial as soundscape interpretation depends on need satisfaction, and different activities entail different needs. As stated by Andriga et al. (2013, p. 2740) “*when stimuli help to satisfy one’s needs one is in control and free to act*,” thus allowing address needs proactively “*before they become uncomfortable and long before they endanger existence*,” leading to a positive evaluation. Conversely, in the case of stimuli that hinder need satisfaction, one becomes aroused, and motivated so as to satisfy the need as soon as possible, thus behaving reactively, and leading to a negative soundscape interpretation (Andringa and Lanser, 2013).

Regarding the impact on WFH, a negative assessment results from the presence of intrusive noise that causes annoyance, distracts and impedes communication in teleworking apps. A similar appraisal is reported amongst relaxation activities that require concentration, such as reading. This type of impact is typically present in work and study environments, as described by the literature on the impacts of noise on cognition (Clark and Paunovic, 2018). The literature on children has put forward pathways of noise effects on cognition, which include impaired reading comprehension and speech perception, impaired attention, learned helplessness, frustration, annoyance, increased arousal which may lead to increased stress, and lower mood and reduced performance (Basner et al., 2014; Clark and Paunovic, 2018).

Loud noises from outside, such as traffic or construction sites, noise from human beings (such as intelligible and non-intelligible speech, impact sounds, music and TV) from people at home, family members, and neighbours were most frequently reported as disruptive elements for homework. In a preliminary analysis on the same dataset (Torresin et al., 2022), people at home were the most frequently mentioned sources of annoyance for home working, followed by traffic, neighbours and construction sites. The concentration of more people in the domestic environment led to overcrowding issues and related noise exposure that negatively impacted WFH. House-related noise annoyance increased compared to pre-pandemic period (Şentop Dümen and Şaher, 2020), becoming the main source of disruption whilst WFH, proportional to the number of persons present at home (Puglisi et al., 2021; Torresin et al., 2022).

Noise from outdoor sources (e.g., traffic, construction sites) and neighbours are other sources of disruption during home working. A previous study reported that 75% of participants were annoyed by noise because it reminded them of the presence of the noise source and prevented them from maintaining desired mental states, whilst 25% reported that salient noises disturbed their activities because they had to pay attention to sounds they did not want to hear, making them the focus of attention (Andringa and Lanser, 2011). Home working in presence of sound stimuli may involve a balance between two aspects of directed attention: *“(perceptual) externally aimed selective attention, which is facilitated by strong or otherwise relevant input, and internally aimed directed attention for mental task performance that requires a precise mental state, unperturbed by perceptual distractors*” (Andringa and Lanser, 2013, p. 1452). This results in additional mental effort associated with the need to repeatedly switch between perceptual distractors and work tasks. In the worst case, a situation arises in which the switching effort becomes so large that it is impossible to perform the task correctly because the distractors have become dominant (Andringa and Lanser, 2013), leading to mental health impacts (Bergefurt et al., 2022a). Besides this “attentional capture” mechanism (Bell et al., 2012), a further mechanism of noise disruption on cognitive resources is through “interference by process,” in which similar cognitive processes are involved in unconsciously analysing background noise whilst consciously tackling a task, thus resulting in conflict and consequent reduced performance (Marsh et al., 2009). The impact on performance and the consequences in terms of stress and mood reduction make WFH the activity most negatively affected by the acoustic environment at home amongst those investigated, followed by relaxation activities (see Figure 5).

As regards noise impacts on leisure activities, as described in previous analyses on the same dataset (Torresin et al., 2022), most frequently mentioned sources of nuisance are neighbours, traffic, and people at home. This is more likely in apartment blocks compared to terraced or semi-detached houses, resulting in higher perceived acoustic saturation of the environment, or “content” (Torresin et al., 2022). Neighbour noise has been a major source of noise nuisance and public complaint for decades in the United Kingdom, and causes a higher percentage of complaints, relative to the number of people who hear it, than road traffic or aircraft noise (Stansfeld et al., 2000). A pre-pandemic study in London reported neighbours’ and external noises perceived inside dwellings to be equally annoying (Lee et al., 2020). During the lockdown, however, the situation was further exacerbated, and neighbours’ perceived noise level, relative annoyance and noise complaints in London increased significantly (Lee and Jeong, 2021; Tong et al., 2021).

The negative interpretation of the soundscape during leisure activities could again be explained by Andringa and Lanser’s (2013) cognitive model describing how the sound content, and related safety and danger indicators, allows freedom over mental states or forces a vigilance state associated to arousal. Therefore, loud sounds (e.g., from neighbours, outdoor sources or housemates) can act as exogenous motivators that compel one to be more alert and/or attend certain sources that can dominate mental states. Moreover, loud noises can interfere with listening to TV or listening at a comfortable volume and spoil the quality of music listening, resulting in an uncomfortable experience.

Activities that involve the generation of sound stimuli (e.g., music and TV during relaxation and training, impactive noises during training and sounds emitted during sexual activity) are accompanied by a fear of disturbing neighbours and housemates. In the case of sexual activities, hearing intrusive noises can make one vigilant and prevent full freedom in mental states. Notably, hearing voices and sounds that imply the presence of other people increases the awareness of being heard and raises privacy concerns. This undermines a fundamental dimension of healthy living, which “*provides a feeling of home, including a sense of belonging, security and privacy*” (World Health Organization, 2018, p. 2), which has also been identified as a perceptual dimension of the indoor soundscape in residential buildings (Torresin et al., 2020a).

In some cases, participants reported that sound stimuli have no impact on their home activities, particularly on physical activity, sexual activities and listening to music (see Figure 5). The lowest influence is therefore for tasks that involve less cognitive effort, which take place at quieter times (i.e., evening or early morning) and that are accompanied by the playing of music or the use of headphones, allowing to mask environmental noise and isolate from the surrounding environment. In some cases, participants reported having learned to ignore background noise whilst engaged in the task. Habituation was identified as a factor moderating the impact of the urban context on soundscape appraisal, leading to a lack of reported impact (Figures 1, 2). Previous research suggests that children chronically exposed to noise learn to “tune out” noise distractors and this ability is stronger during the first few years of exposure (Evans and Lepore, 1993). However, mixed results are found in the literature as regards to habituation effects, the degree of habituation is never complete and differs from individual to individual (Marquis-Favre et al., 2005; Basner et al., 2014; Torresin et al., 2019a). Habituation might be interrelated with other personal and demographic traits (e.g., noise sensitivity, gender and sex), which are reported to have a potential influence on acoustic perception (Marquis-Favre et al., 2005). In quantitative analyses on the same dataset (Torresin et al., 2022), noise sensitivity was actually found to moderate the relationship between perceived sounds and perceptual outcomes (comfort and content) and people’s wellbeing. Higher perceived content (i.e., busier acoustic environment) was associated with female participants, and higher acoustic comfort whilst WFH was related to elder participants (Torresin et al., 2022).

In case of more cognitive demanding tasks involved in WFH, a selective attention process may occur, which allows to “block out” noise distractors (Halin et al., 2014; Caviola et al., 2021). Finally, minimising the impact of noise could be the outcome of a “denial/avoidance” coping strategy, such as pretending that noise is not present or that it is not annoying (Stansfeld et al., 2000). This would lead to an underestimation of the impacts of exposure to sound stimuli at home.

The soundscape perspective also made it possible to highlight the positive effects of exposure to pleasant sounds in the home environment. A quiet environment is conducive to concentration and relaxation, allows intelligibility in work communications and listening to music and TV at a comfortable volume, without having to overpower background noise. According to Riedel et al., a calm environment enables easy assessment of acoustic safety and leaves ample mental resources available for proactive activities such as caring for oneself or others (Riedel et al., 2021).

Natural sounds are amongst the most desired sounds at home, for their perceived ability to enhance positive perception, reducing stress, improving mood and cognitive performance. Those findings are consistent with the Attention Restoration Theory (Kaplan, 1995) and large part of recent literature on the effect of auditory experience of nature on people (Ratcliffe, 2021).

Urban sounds, sounds from other family members or housemates, even noise from building services can be beneficial to create a sense of place and provide indications of safety. Feeling in contact with the external environment and comforted by hearing the sounds of the family are aspects that are often stressed in the participants’ words in relation to the particular lockdown condition, where those stimuli provided important support and companionship whilst carrying out activities.

Perceived control over the environment is a key factor that moderates the influence of the acoustic environment on soundscape interpretation (Figure 1). The construct of perceived control is central to the literature on indoor environmental quality (Hellwig, 2015) and is a perceptual dimension underlying affective reaction to indoor residential soundscapes (Torresin et al., 2020a). As reported by Stansfeld et al. (2000, p. 70), “*individuals with an external locus of control are those who attribute outcomes to the actions of others, whereas individuals who believe that outcomes are determined by their own behavior have an internal locus of control*.” Participants recognise that sources over which they have no control (e.g., neighbours) are judged more negatively than those over which they do have direct control, such as music. The former induce frustration and a sense of helplessness, whilst the latter allow to shape the soundscape to their liking, leading to sensory pleasure.

Self-selected music and other multimedia content (radio, podcasts, TV) is often mentioned as a welcome sound stimulus, particularly during relaxation and workout activities. As also confirmed in the literature, music can improve the psychophysical state during home exercising (Fallis, 2013), reduce stress (Pelletier, 2004) and support work performance by increasing positive affect (Lesiuk, 2005).

The presence therefore of music and natural sounds, or sounds of the city and family can contribute to a lively and engaging soundscape, i.e., an acoustic environment that is both safe and interesting and stimulates learning and active involvement (Riedel et al., 2021).

#### Coping strategies

When the result of soundscape appraisal is dissatisfaction, this would motivate active coping behaviours or resignation (Wierzbicka et al., 2018). Coping strategies were described within the third theme of the thematic analysis. These include controlling doors and windows, playing music and turning up the volume as masking strategy, wearing headphones not to disturb or to improve listening quality, and switch to noise-cancelling ones. If these immediate strategies are not sufficient to restore satisfaction, behavioural changes may be adopted: moving to a different room and/or shifting the activity at quieter times, muting work calls, changing the type of sport activity so that it does not involve jumping on the floor, or sound-constraining sexual activity.

Perceiving the environment as incongruent with one’s needs and resignation trigger a stress response that can lead to negative health effects. Active behaviour has the potential to reduce stress. However, if wrongly targeted, it can also create side-effects that in turn impact on health (Wierzbicka et al., 2018; Torresin et al., 2020b). The reproduction of sounds for masking purposes may lead to prolonged exposure to harmful noise levels. Moreover, noise-induced modification of working patterns, home workout and sexual activities can lead to a reduction in work performance, impair sexual wellbeing, and physical activity, aspects that are central to people’s wellbeing and thus of the concept of healthy building.

### *Desiderata* in the ideal indoor soundscape and implementation in the design of the post-pandemic housing

The qualitative analysis of verbal material provided by participants made it possible to identify the “ingredients” that characterise the ideal indoor soundscape for the various activities investigated (Table 6). Findings will be discussed in the following in order to derive indications for research and practise on housing design.

#### Sound insulation, freedom of sound expression, and the need for quietness

Amongst the aspects most often mentioned, is the demand for a “quiet” environment, especially in relation to relaxation activities, home working, and sexual activity, as shown in Table 6. This demand is often accompanied by the request to be free to generate “noise” at home, without the fear of being heard (e.g., privacy concern during sexual activities) or disturbing others (e.g., whilst playing music or performing physical activity). All these aspects lead to the demand for higher airborne and/or impact sound insulation performance of floors, walls, façades and windows. This is common to other studies performed during the pandemic, in which higher insulation ranked amongst the most desired factors after the request for more outdoor spaces (Alonso et al., 2021) or better visual quality from home (Zarrabi et al., 2021). Similarly, in a survey conducted in Italy, 49% of the participants stated that they would prefer a slightly quieter environment (Salamone et al., 2021), whilst data derived from an international survey showed that 30% of participants wanted quieter urban noise and 46% wanted more quiet from indoor noise during the confinement period (Caniato et al., 2021).

Whilst expressing a request for “quietness,” in the present study participants often specified they would not want a “silent” environment and emphasised the importance and meaning associated with positive sound stimuli. This is in line with what has been reported in the soundscape literature regarding quietness definition, which would not only or necessarily be related to a dB sound level reduction, but rather a more complex construct, in which pleasant sounds and (multi-sensory) safety indicators would play a key role (Andringa and Lanser, 2013; Kang et al., 2016; Aletta and Kang, 2019).

If quietness also depends on the quality of available sounds, it follows that sound insolation, particularly façade sound isolation, should be ideally “selective.” Unlike sounds from neighbours, which are rarely appreciated (Frescura and Lee, 2022), outdoor sounds can be both pleasant and unpleasant. Therefore, building façades should be ideally able to selectively filter out noise and let in sounds, and to be tuneable according to changing activities and needs. This therefore leads to rethink the role of window opening and ventilation as a tool able to “*transmit, block or adjust outdoor sounds to provide a connection with the outside, release wanted sounds, or mask unwanted ones*” (Torresin et al., 2019b, p. 17). Indoor-soundscape-informed window automations (Torresin et al., 2019b; Fusaro and Kang, 2021), active noise control technologies applied to façade openings (Lam et al., 2021), and metamaterial design (Fusaro et al., 2022) could make this possible in the future.

As a further aspect, findings highlighted the importance of sound insulation to allow freedom of sound expression in domestic settings and to build a sense of privacy, without placing noise-related restraints on relaxation, home workout and sexual activities. This aspect is often overlooked in the literature but may lead to activity modification or, eventually, to giving it up, resulting in reduced opportunities for restoration, physical activity and compromised sexual wellbeing, with consequent degradation of health opportunities at home.

#### Urban planning, nature-based solutions, and indoor soundscape

The results of the present study reinforce the need for soundscape-conscious urban design to ensure a positive indoor soundscape at home, as already highlighted in a previous interview with experts on salutogenic acoustic design (Torresin et al., 2020b).

A first way in which urban planning can contribute to a positive soundscape is by ensuring quiet sides of buildings. A previous structural equation modelling on the same dataset showed how the availability of a quiet side was associated with higher comfort whilst working and relaxing at home (Torresin et al., 2022), in keeping with literature findings on the beneficial effects in terms of reduced annoyance, increased health and quality of life (Kluizenaar et al., 2011; Van Renterghem and Botteldooren, 2012; de Kluizenaar et al., 2013; Van Kamp et al., 2016).

As previously discussed, the quality of sounds that compose the acoustic environment would be crucial in building quietness perception. Natural sounds are often reported to be a prerequisite for quietness and resulted to be the most desired sounds in an ideal soundscape, especially in relation to home working and relaxation (see Table 6). Natural sounds are often desired (Axelsson et al., 2010; Torresin et al., 2020a), and can bring restorative benefits (Kaplan, 1995; Ratcliffe, 2021; Uebel et al., 2021). However, their availability in indoor built environments can be challenging. If scepticism accompanies the adoption of artificial natural soundscapes and research to this end is not yet conclusive (Torresin et al., 2020b), the access to natural sounds through ventilation openings can be effective (Torresin et al., 2019b, 2020a). However, this assumes the presence of green areas and a wider adoption of nature-based solutions (NBS). According to the European Commission, NBS are “*solutions that are inspired and supported by nature, which are cost-effective, simultaneously provide environmental, social and economic benefits and help build resilience*” and “*bring more, and more diverse, nature and natural features and processes into cities, landscapes and seascapes, through locally adapted, resource-efficient and systemic interventions*” (European Commission). They include the provision and restoration of green areas, such as parks, urban forests, street trees and green roofs, rain gardens, wetlands, and community gardens (Geneletti et al., 2022). As previously highlighted in the literature, the benefits of NBS on human wellbeing from an improved soundscape are poorly identified and often not taken into account (Dick et al., 2020), or their consideration is limited to the benefit related to noise pollution reduction (European Commission, 2021; Jiang et al., 2022), intended as dB noise level abatement. Therefore, despite the potential to improve people’s wellbeing and quality of life, the benefits associated to NBS in terms of positive soundscapes and improvement of the quality of heard sounds are generally neglected. If included amongst the full suite of NBS advantages, this could lead to greater strength in the adoption of NBS by policy makers and urban planners.

Moreover, whilst the connection to natural sounds has long been a topic of investigation and appears to be important for the perception of quietness and for stress reduction, the literature call for more research on urban sounds potential of defining positive everyday indoor soundscapes (Ratcliffe, 2019; Torresin et al., 2020b). Notably, the present study provides evidence on the positive contribution that sounds from the domestic (e.g., family members) or urban environments (e.g., traffic hum) can make in terms of support, motivation, safety, and connection. This would support the hypothesis that below a certain noise level threshold - which should be defined as a function of the performed task, control opportunities and exposure time, as to prevent health risks - the quality of the indoor soundscape quality would be more related to sound type than to dB noise level value, as discussed before (Torresin et al., 2020b).

#### Activity-based acoustic design at home

The present study has highlighted different acoustic requirements and different mechanisms by which the acoustic environment impacts on activity performance and wellbeing depending on the task at hand. Even if not within the scope of the study, acoustic complaints were often accompanied by the lack of adequate space or sharing available space with other people, who are often engaged in different activities. Previous quantitative analyses on the same dataset highlighted negative impacts on acoustic comfort due to smaller houses and a higher number of people at home (Torresin et al., 2022). The presence of different acoustic requirements for different activities, the performance of new activities at home since the pandemic, and the coexistence of people within the same space make the domestic environment increasingly similar to an open plan office, thus sharing (some) critical issues and design solutions.

Previous studies based on life experiences during the pandemic have reported the need for a dedicated working space at home (Org et al., 2021; Awada et al., 2021b; Bergefurt et al., 2022a,b). Findings from a London-based study lead to recommending the establishment of minimum space standards and the inclusion of “*a wider range of home uses and household compositions in housing provision and design standards to cater to demographic shifts, for example, a growing ageing population, a rise in single-person homes, and an increase of non-related adults living together*” (Alonso et al., 2022, p. 11). Taken together, the provision of adequate space and availability of rooms with different acoustic characteristics depending on the activities performed would be ideal in the case of shared housing. This would lead to activity-based flexible housing, in which people are free to move across different locations matching the requirements of different kinds of activities, as in the case of activity-based flexible offices (Wohlers and Hertel, 2017).

When not possible, the use of (noise-cancelling) headphones, active sound reproduction systems and acoustic shields can help adapt and separate shared environments and make it more flexible for different uses.

### Limitations

Results presented in this study should be interpreted considering certain limitations. The investigation was conducted in winter, and some of the patterns illustrated (e.g., those involving window operation) cannot be generalised to other seasons, given the potential seasonal effects on window opening behaviour reported in the literature (Andersen et al., 2013). The study is qualitative in nature and does not include objective measures of the acoustic environments. Relationships between contextual and acoustic variables and the impacts on activities, are *theorised* or *proposed*, based on participants’ experiences in their own words (i.e., subjectively captured), collected through an online, unsupervised data collection method. Impacts of the sound environment might not be correctly understood, and reported by individuals, thus leading to under-or over-estimation. Personal variables (e.g., gender, age, noise sensitivity) were not included in the qualitative analysis. However, unlike previous quantitative analyses on the same dataset (Torresin et al., 2022), the focus here was on highlighting patterns of influence for the different activities. We expect that individual traits are likely to change the magnitude of these cause-effect patterns (Torresin et al., 2022), not the general mechanisms involved. Future research could use interviews to collect more in-depth qualitative responses coupled with binaural monitored data of the acoustic environment, useful to derive more specific design requirements. In order to reduce the number variables involved, the study focused at this stage on adult participants with self-reported “normal” hearing. However, given the range of auditory conditions and perceptual disorders that affect a relevant part of the population, future investigations should focus on a more aurally diversified sample (Drever and Hugill, 2021). Moreover, we could not at this stage quantify the magnitude of impacts in terms, for instance, of health or performance effects, or effects due the peculiar conditions of the pandemic. Future longitudinal and epidemiological studies may help to better quantify impacts in post-pandemic conditions, across different seasons.

## Conclusion

Taking an activity-based perspective, the qualitative analysis of verbal material collected during the COVID-19 lockdown in London helped to describe (1) positive and negative impacts of the “actual” acoustic environment at home, and (2) the features of an “ideal” soundscape for work, relaxation, workout, and sexual activity at home.

With regard to the first objective, taking the framework provided by ISO 12913-1 as reference, the thematic analysis led to the identification of four themes concerning contextual features, the characteristics of the acoustic environment, soundscape interpretation, and coping strategies, together with their interrelationships. Within the acoustic environment, the main variables were related to the type of sound (e.g., neighbours’ noise, family members, music), sound dominance (i.e., loudness, quietness), and temporal patterns (e.g., impulsivity). The range of impacts varies from no effect on task execution, to disruption, distraction, concern of disturbing others or being heard, to support of concentration, relaxation, motivation, freedom of sound expression, feeling of being connected to the surroundings and comforted by the sounds of others, according to mechanisms described in the study. Negative appraisal can generally trigger coping strategies (e.g., controlling windows, playing music, wearing headphones) and behavioural changes (e.g., lowering the volume of the voice or music, muting oneself during call, changing workout type) that can in turn limit or enhance the freedom of behaviour, affect or foster wellbeing.

Negative impacts were most frequently reported towards WFH (by 55% of the participants), followed by relaxation activities (40.6%), sexual activities (30.1%), and home workout (20.1%). No impact resulted especially in relation to home exercise (by 55.8% of the participants), and sexual activities (55.2%), and to a lesser extent to relaxation activities (39.0%) and home working (24.2%). In general, sound stimuli can be particularly disruptive and frustrating for tasks requiring concentration and intelligibility (e.g., reading, WFH, yoga and meditation), which do not offer masking opportunities through sound produced during the execution of the activity itself (e.g., by playing music or watching TV) or which generate noise that may cause disturbance to others (e.g., playing loud music) or embarrassment and privacy concerns (e.g., during sexual activities).

The ideal soundscape is described by a quiet, well-sound insulated environment, which guarantees privacy, intimacy and freedom of sound expression, but also access to positive sounds (i.e., natural sounds, music, a soothing urban background). The study complements literature findings on housing design directions in light of the COVID-19 pandemic consequences, and highlights the need for:

- Adequate sound insulation of building components not only against external and neighbouring noise but also to allow activities to be carried out without noise-related constraints
- Use of nature-based solutions to access quiet urban areas and bring positive soundscapes indoors through building facades
- Increased space standards coupled with an activity-based design of dwellings, taking into account the varied uses and needs of contemporary housing, and the varied household compositions.

## Data availability statement

The raw data supporting the conclusions of this article will be made available by the authors, without undue reservation.

## Ethics statement

The studies involving human participants were reviewed and approved by the research was approved *via* the UCL IEDE Ethics departmental low-risk procedure on November 26th, 2020. The patients/participants provided their written informed consent to participate in this study.

## Author contributions

ST, FA, FB, RA, TO, and JK designed the study. ST and FA conducted the analysis with input from ER. All authors have seen and approved the final version of the manuscript for publication.

## Funding

This work was funded by the Chartered Institution of Building Services Engineers (CIBSE) within the project “Home as a place of rest and work: the ideal indoor soundscape during the COVID-19 pandemic and beyond.” FA was partially supported by the UCL Health of the Public Small Grants scheme (project: DeLTA, 2020–2021).

## Conflict of interest

The authors declare that the research was conducted in the absence of any commercial or financial relationships that could be construed as a potential conflict of interest.

## Publisher’s note

All claims expressed in this article are solely those of the authors and do not necessarily represent those of their affiliated organizations, or those of the publisher, the editors and the reviewers. Any product that may be evaluated in this article, or claim that may be made by its manufacturer, is not guaranteed or endorsed by the publisher.
